# Regardless of personality, males show similar levels of plasticity in territory defense in a Neotropical poison frog

**DOI:** 10.1038/s41598-023-30546-7

**Published:** 2023-03-01

**Authors:** Mélissa Peignier, Lauriane Bégué, Max Ringler, Birgit Szabo, Eva Ringler

**Affiliations:** 1 Division of Behavioural Ecology, Institute of Ecology and Evolution, University of Bern, Wohlenstrasse 50a, 3032 Hinterkappelen, Switzerland; 2 Institute of Electronic Music and Acoustics, University of Music and Performing Arts Graz, Graz, Austria

**Keywords:** Behavioural ecology, Animal behaviour

## Abstract

Animal personality traits are sometimes linked to an individual’s degree of plasticity, with certain personality types being more plastic than others. In territorial species, consistently high levels of aggression might increase the risk of harmful fights, while consistently low aggression might lead to the loss of a territory. Consequently, reacting plastically with an appropriate territorial response should be beneficial to avoid these risks. An integrative investigation of both personality traits and plasticity can help us better understand the dynamics of aggressive interactions during male-male competition. Here, we used a free-ranging Neotropical poison frog population to investigate the role of plasticity in male territorial aggression towards intruders. We conducted repeated standardized territorial intrusion experiments mimicking frogs of different body sizes via playback calls with different peak frequencies. We found individual repeatability for the latency to reach and approach a simulated intruder and observed that both aggressive and less aggressive males decreased their level of aggression towards big intruders. However, our results do not support a correlation between personality and plasticity in the context of male territory defense during the breeding season. We discuss how such a correlation between personality and plasticity might be conditional on the costs and benefits across contexts.

## Introduction

Individual animals often show within-individual consistency and between-individual variation in their behavioral responses across time and/or context—also termed ‘animal personality’^1–4^. Individuals who are very predictable in their behavior are typically assumed to be limited in how much they can adjust their behavior to different contexts (i.e., “behavioral plasticity”). However, several studies have shown that the existence of personality does not preclude behavioral plasticity^5,6^. When personality and plasticity are correlated, individuals may be constrained in the direction and strength of behavioral changes they can exhibit when responding to changes in their environment^7–10^. However, personality and plasticity can also coexist without being correlated, in which case all individuals will react plastically in a similar direction regardless of their personality. The use of a behavioral reaction norm framework can be used to quantify such behavioral variation, by measuring a single trait across several contexts^11^.

Animal personality is typically characterized along different axes (e.g., aggressiveness, exploration, boldness, activity, and sociability)^12^, and individuals can take any values along these axes. For example, individuals showing a proactive personality type are typically more risk-taking with higher levels of aggression compared to reactive individuals. Proactive individuals also often rely more on routines (i.e., ‘unresponsive’ individuals) and are better adapted to stable conditions, while others will respond plastically to environmental cues^9,10,13^ which can result in differences in plasticity across personality types. For instance, less aggressive mice (*Mus musculus*) adjust their level of aggression according to social context while highly aggressive mice are more rigid^14^. Such differences in the level of behavioral plasticity can originate from constraints on the morphology, physiology or cognitive abilities of an individual^15–17^, or from differences in life history strategies^18^.

Animal personality can influence reproduction and survival by, for example, affecting male-male competition, dispersal, or foraging efficiency^19–21^. Aggressiveness plays a particularly important role in territorial species in which more aggressive individuals are often more successful in acquiring and/or defending a territory, but more at risk of engaging in harmful fights^22^. Therefore, reacting with the appropriate level of aggression to a potential competitor is important. An individual showing consistently high levels of aggression will incur a cost when attacking stronger competitors, while an individual showing consistently low levels of aggression will be more likely to lose their territory. In addition, the presence/absence of certain resources (e.g., food patches, mating partners, etc.) might alter the costs and benefits linked with behavioral consistency and thus how an individual responds towards intruders. Integrating animal personality and plasticity can help better understand the dynamics of aggressive interactions during male-male competition.

Neotropical poison frogs (Dendrobatidae, sensu^23^) show prominent territorial aggressiveness which can be easily elicited experimentally. *Allobates femoralis* is a poison frog which acoustically establishes multi-purpose territories during the prolonged breeding season^24–28^. In this species, territories serve for feeding, advertising, courtship, and mating^29^, and are defended during the entire breeding season, whether the male has clutches or not. The reproductive success of males is directly linked to their ability to hold a territory^28^, and previous studies have confirmed that males consistently differ in their level of territorial aggression and fall along a continuum of aggressiveness^30,31^. Specifically, the personality trait aggressiveness can be seen as a latent variable that can be derived from the covariance between the latency to turn and jump towards an intruder and the speed to approach the intruder^30^. Like in many frogs, *A. femoralis* call peak frequency^32^, but not sound pressure level^33^, is negatively correlated with, and therefore indicates, body size.

In this study, we investigated how personality, especially aggressiveness, is linked to behavioral plasticity (i.e., how much an individual can adapt its behavior to different contexts) in the level of territorial aggression in *A. femoralis*. We repeatedly measured behaviors expressed by individuals defending their territory against an average sized intruder, using standardized experiments to assess the individual’s personality along the aggressiveness axis. In subsequent trials we then mimicked intruders of different body sizes via playback calls with low and high peak frequencies to investigate the direction and strength of individual behavioral changes in response to modified agonistic encounters. Based on our knowledge of the study species, we expected three possible outcomes:

1. all individuals show a high level of territorial aggression towards any intruder to avoid the high costs of losing a territory (cf.^28^);
2. all individuals show plasticity if the costs associated with fighting an overpowering opponent are higher than the costs associated with immediately withdrawing and establishing a new territory;
3. if personality is linked to plasticity, males at one extreme of the aggressiveness personality axis (e.g., low aggressive) adjust their level of aggression to intruders of different sizes, while males at the other extreme (e.g., highly aggressive) are less responsive and rely on routines, showing similar levels of aggression regardless of intruder size.

## Methods

### Ethics

This study was approved by the scientific committee of the ‘Nouragues Ecological Research Station’. Sampling for this study was conducted in strict accordance with current French and EU law. We followed the guidelines laid out by the ASAB for the treatment of study animals and Teaching^34^ and the ARRIVE guidelines^35^. Research permits were provided by the CNRS Guyane (‘Centre National de la Recherche Scientifique Guyane’), by the ‘Ministere de la transition ecologique et solidaire’ (permit number: TREL2002508S/303), and the Secretariat of the Convention on Biological Diversity (APA declaration: TREL1734890A/34).

### Study site

We used a free-ranging experimental population of *A. femoralis* that was introduced in 2012 on a small (~ 5 ha) river island in tropical lowland rainforest located in the Nouragues Nature Reserve, French Guiana (4°02′ N 52°41′ W;^36–38^). The study was conducted between April–May 2022 during the reproductive period of the species^25^ and all tests were performed during its main calling activity (1400–1900 h,^39^). We surveyed the population every day aiming to sample all adult males present on the island and caught frogs using a transparent plastic bag to minimize direct contact and stress. We took dorsal photographs on mm-paper to determine body size (snout urostyle length) using the software Image J 1.52a^40^, and ventral photographs to identify frogs via their distinct belly patterns. We later confirmed identifications from the field with the pattern matching software Wild-ID^41^. In total we caught 76 males with an average body size of 2.9 cm (± 0.1 SD).

### Territorial defense test

**Figure 1:**
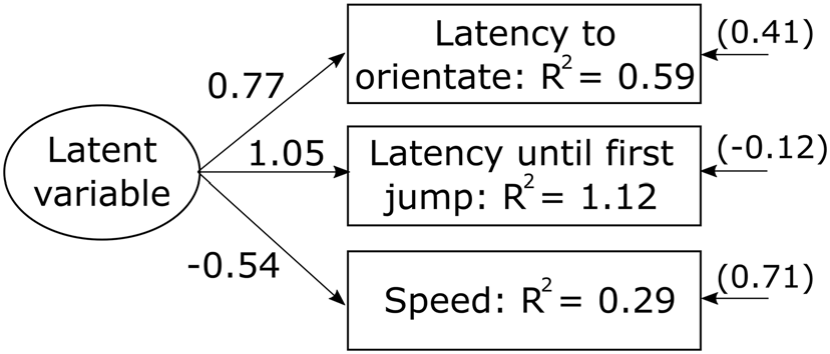
Path diagrams of the structure equation model explaining the covariance structure among the three behaviors assessed during the territorial defense test. R^2^ represent the variances of the different behaviors. Numbers associated with arrows represent how behavioral responses are predicted to change based on changes to the latent variable. Number in brackets represent error variances associated to each behavior.

To assess individual levels of consistency and plasticity in aggressiveness, we used acoustic playbacks of advertisement calls simulating an intruder to induce territorial defense behavior in the focal territory holder^26,42^. Advertisement calls consist of four notes each sweeping up in frequency and ranging between 2.5 and 4.1 kHz^24,42,43^. Territory holders typically respond to calling intruders by orientating their head/body and jumping towards the intruder. If an intruder does not retreat and continues calling, the territory holder might escalate the conflict and start wrestling with the intruder^26,42,44–46^. We used a loudspeaker with an integrated music player (MUVO 2c, Creative, Singapore) for playbacks. We centered the loudspeaker on top of a black PVC disc (radius = 15 cm) that was positioned with a laser rangefinder (DLE 50, Bosch, Stuttgart, Germany) at 2 m from the focal male (cf. set-up shown in Fig. 1 in^31^). The experimenter stood another meter behind the setup and recorded the behaviors of the focal male (head-body orientation, jump, touching the disc) using a digital voice recorder (ICD-PX333, Sony, Tokyo, Japan). Each focal male was given 30 s acclimatization time to resume normal behavior after installation of the setup. We ended the trial when the focal male touched the disc or when the playback ended after 5 min. We excluded trials from our analysis if the focal male started to call, as this is an indication that he assumed the simulated intruder to be outside his defended area (cf.^24,26^).

To assess baseline levels of aggressiveness, we first tested individual frogs multiple times with signals mimicking an ‘average sized intruder’ (mean = 3.11 repetitions per individual, range = 1–4). We created our playback signals using an artificial call from previous studies on *A. femoralis,* featuring the average spectral and temporal parameters of *A. femoralis* in the Nouragues Reserve (i.e., ‘standard call’ of^24,43,44,47^). We created six further variations in addition to the ‘standard call’, with different temporal properties (inter-note and inter-call intervals) within the natural variation. To prevent habituation, for each baseline trial, we randomly used one of the seven different calls.

Subsequently, we assessed individual responses to a low and a high frequency call in random order (i.e., one test per individual per condition). These two conditions both used the ‘standard call’, but we changed the pitch of the signal to alter the peak frequency by ± 3 SD (≙ 10.97%, i.e., peak frequency of the high frequency call = 3778 Hz, low frequency call = 3022 Hz). Thereby we created two stimuli close to the extremes of the natural peak frequency range of the species (2900–3900 Hz) that has been established in previous studies^42,43,48^). Following the common assumption of a (quasi) linear relationship between call frequency and body size within a given species [see^43,49^ for *Allobates*,^50^ for tree frogs,^51^ for frog in general] our signals mimicked a particularly large (low frequency call) and a particularly small (high frequency call) intruder at the both extremes of the natural size variation.

All signals were played from WAV-files (16-bit, 44.1 kHz) with the same volume setting resulting in an SPL above 56 dB at the location of the focal frog, which is the minimum necessary to elicit a behavioral response in territorial males^47^. Individual frogs were never tested twice within at least 24 h to avoid habituation to the synthetic signals (cf.^30^). To compensate for local acoustic conditions in our analysis, we measured the SPL (re 20 µPa, dBA, fast) of the respective test signal at the location of the focal frog after the trials, using an SPL meter (SL-100, Voltcraft, Hirschau, Germany).

### Data processing

File names of the voice recordings were blinded. We extracted the latency to orientate (first head-body orientation, in s), until the first jump (in s), and calculated the speed to reach the speaker (in cm/s). A previous study in the same population found that latency to orientate, latency until the first jump, and speed to reach the intruder are repeatable and that the personality trait aggressiveness can be derived from the covariance between these behaviors^30^ and the corresponding analysis of our data corroborated these findings (see “Statistical analysis”). We assigned a censored value of 0 cm/s for the speed of males who did not reach the speaker, and a censored latency of 300 s (5 min = total duration of each trial) to males who did not perform a head-body orientation or a jump.

### Statistical analysis

We confirmed that male *A. femoralis* showed consistent between-individual differences in aggressiveness by estimating the repeatability of each behavior (i.e., latency until to orientate and until first jump, speed to reach the speaker) using the ‘rptR’ package^52^. We used the function “transformTukey” to apply a constant transformation on the response variables to fit a normal distribution and considered behaviors to be repeatable if the 95%-confidence interval (CI) did not overlap zero. We estimated repeatability from models fitted with a Gaussian error distribution for all three transformed variables and included ID as a random effect in all models. For this analysis, we only used individuals who were tested at least twice in the territorial defense test (N = 254 trials on 51 individuals).

We further confirmed the structure of behaviors into functional units as found in^30^. To do that, we investigated the phenotypic covariance structure among the different variables measured during the territorial defense tests. We used the SEM package and applied structural equation modelling to the phenotypic covariance matrix derived from the means of each behavior for each individual, in order to avoid pseudo-replication^53^. We built a model with a latent variable explaining the covariance between the three measured behaviors and compared it to a null model based on differences in Akaike’s information criterion (AIC) values. Small values indicating higher parsimony and a ΔAIC ≥ 2 indicating significant differences^54^.

To investigate if focal males adjusted their behavioral responses according to their aggressiveness levels when encountering average, big, and small intruders, we performed a random regression analysis^55^ and fitted three models: for the (1) latency to orientate, (2) latency until the first jump, and (3) speed to reach the setup (response variables). ‘Treatment’ (average, big, small intruder) was included as a fixed effect^55^. For each transformed response variable, we compared a random intercept (individual identity) model to a random intercept and slope model (intercept: individual identity, slope: ‘treatment’). We added body size as another fixed effect to account for variations in the perceived size difference between the focal male and the big/small intruder. We used a Bayesian approach^56^ and fitted the models using the package brms^57^. We compared model fit using the widely applicable information criterion (WAIC^58^) and selected the best fitting model, with smaller values indicating higher parsimony^55,59^. For all models we used an uninformative prior and ran two Markov chains with 3,000 iterations each and a burn-in of 500. We selected every 2nd posterior parameter sample after the initial burn-in^55^. We present estimates and confidence intervals generated from the best fitting models. We made sure that our models converged by ensuring that Rhat parameters were close to 1^55^. All statistical analyses were performed in R v3.6.0^60^.

## Results

We repeatedly tested 64 males with playbacks mimicking an averaged size intruder resulting in a total of 199 trials (mean = 3.11 repetitions per individual, range = 1–4). Of those 64 males, 34 were also tested with both a playback simulating a small intruder and a playback simulating a big intruder. The other 30 males who were not tested were either not found again, or we did not manage to test them with the small/big intruder playback calls within the duration of the field season. On average, consecutive trials with the same individual were 5.36 ± 4.59 SD days apart.

We found similar results as^30^ regarding repeatability and covariance structure of the behaviors measured in the territorial defense test. Both the latency to orientate and the latency until the first jump had a repeatability of R = 0.23 (first head-body orientation: p < 0.001, 95%-CI 0.11–0.36; first jump: p < 0.001, 95%-CI 0.06–0.32). The speed to reach the speaker had a repeatability of R 0.40 (p < 0.001, 95%-CI 0.25–0.53). The best SEM model supported a latent variable explaining the covariance of the three behaviors measured (Fig. 1, ΔAIC between full and null model = 88.2).

**Figure 2:**
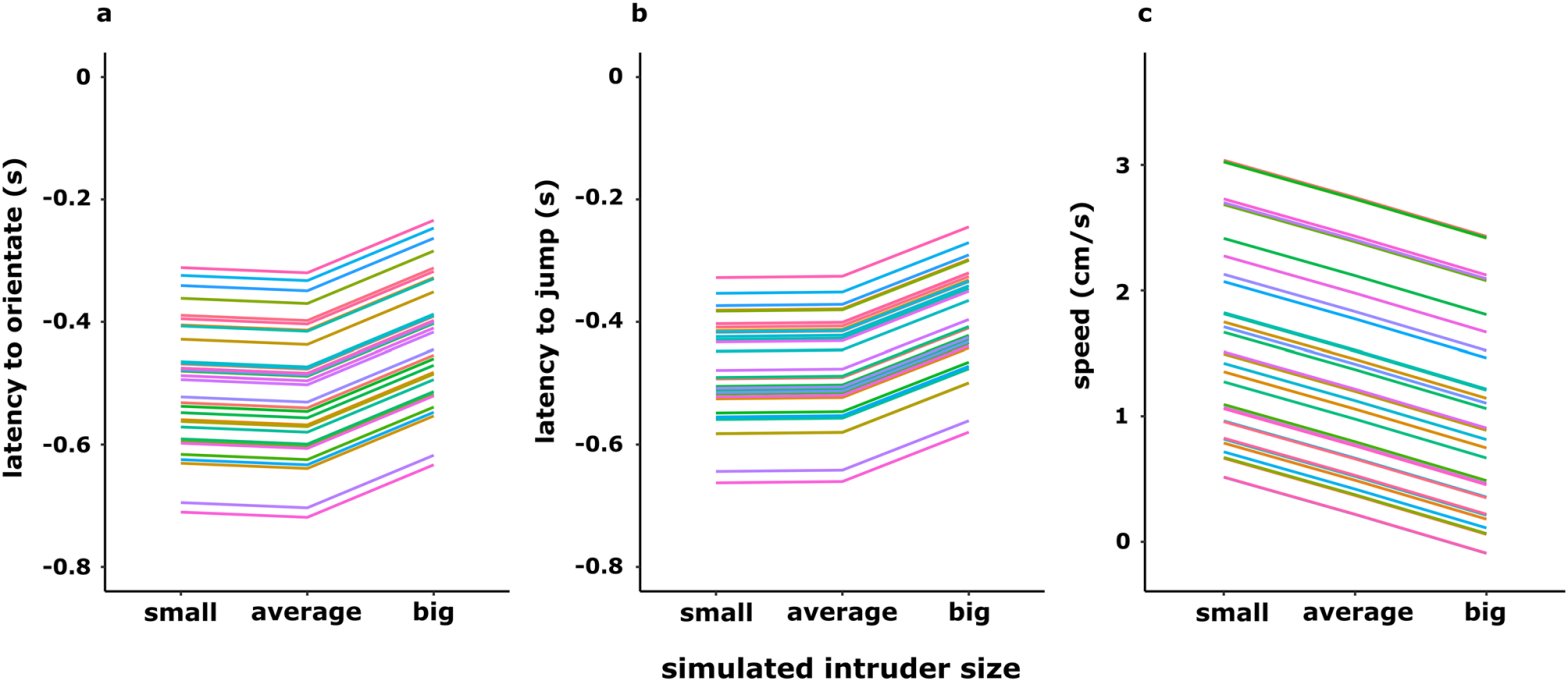
Predicted individuals’ latency to orientate (**a**), latency to jump (**b**) and speed to approach (**c**) a small, averaged sized, and big intruder. Each panel presents the prediction lines of the best fit model (random intercept model) which assumes that males do not differ based on their aggressiveness level in how they adjust their behavioral response across the three treatments. Each line represents a different individual. A constant transformation has been applied on the three response variables.

**Figure 3:**
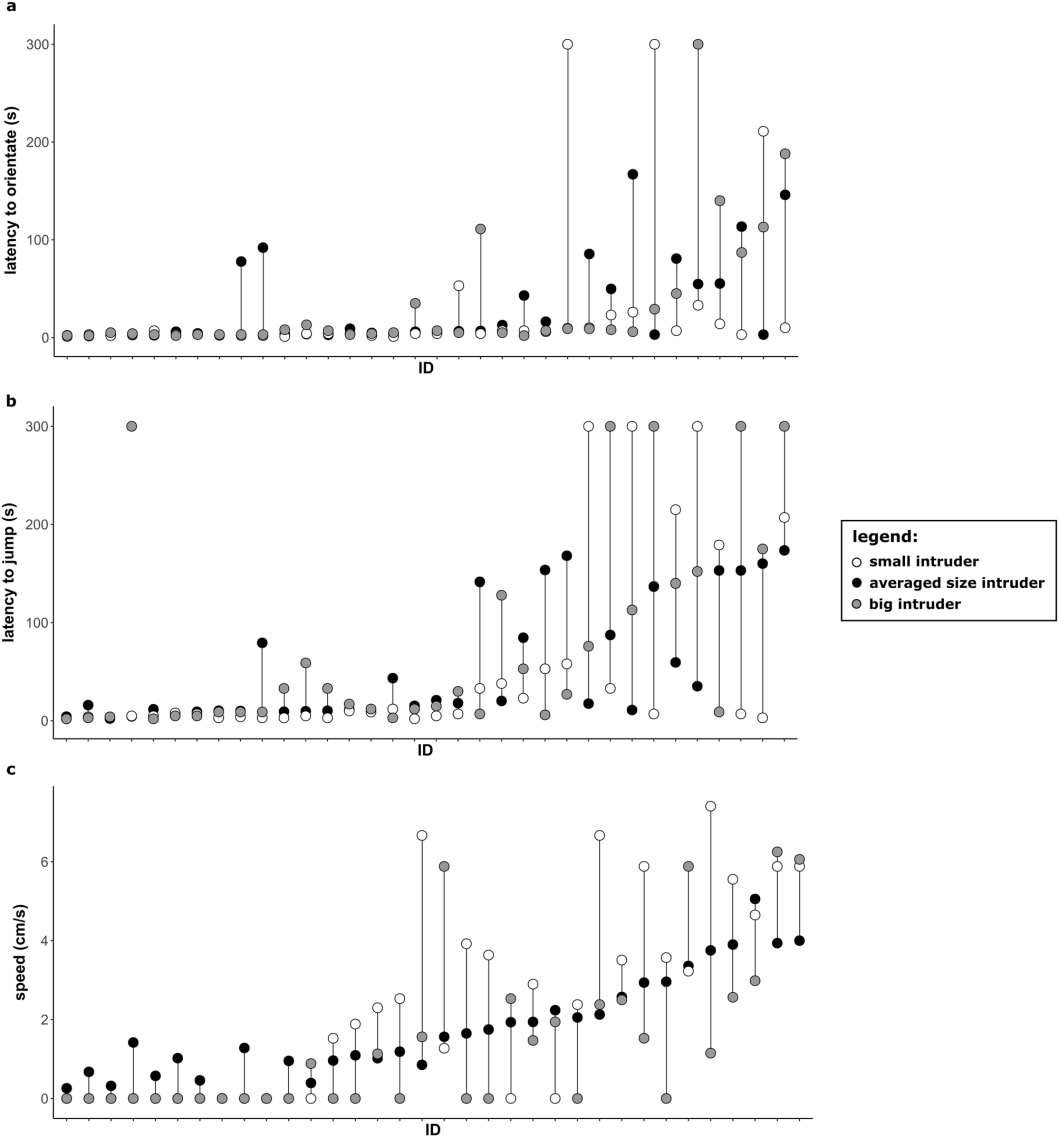
Individual responses towards differently sized intruders (white dots—small intruder; black dots—averaged size intruder; grey dots—big intruder). Black dots are average of the values measured in the four baseline territorial defense tests. (**a**) latency to orientate towards the intruder, (**b**) latency to jump towards the intruder, (**c**) speed approaching the intruder. Results for the 34 males that were tested with the three types of intruders are presented. Males are ordered by their median. The vertical lines are visual aid to show which dots belong to which individual.

In the analysis of the three different peak frequency treatments, the simpler model including only a random intercept always fitted the data best (model (1) for the latency to orientate: ΔWAIC = 1.37, model (2) for the latency until the first jump: ΔWAIC = 0.58, model (3) for the speed to reach the set-up ΔWAIC = 4.75), suggesting that plasticity was equal across aggressiveness levels. We only found moderate evidence for an effect of treatment on the speed to approach an intruder, as credible intervals slightly overlapped zero. Overall, individuals were slower to approach (95%-CI − 0.66, 0.02) big intruders. We found strong evidence that treatment affected the latency to orientate and jump towards an intruder, as individuals were slower to turn (95%-CI 0.01, 0.19) and jump towards (95%-CI 0.01, 0.15) big intruders (Table 1, Fig. 2). Individuals did not differ in their behavioral responses towards small and average sized intruders (Table 1, Fig. 2). The focal individual body size, and therefore the resulting presumable size difference between the focal male and the big/small intruder, did not influence its reaction towards the simulated intruder (Table 1). The individual-based graphs (Fig. 3) show that the latency to react to a big intruder was almost always higher compared to the latency to react to a small intruder.

**Table 1:** Results of the Bayesian random regression models fitted with a random intercept (for the individual).

Response variable		Estimate	Estimate error	l-95%	u-95%
Latency to orientate	*Group-level effects (ID)*				
	sd(intercept)	0.12	0.02	**0.08**	**0.18**
	*Population-level effects*				
	Intercept	0.00	0.60	− 1.17	1.22
	High frequency call	0.01	0.04	− 0.07	0.08
	Low frequency call	0.09	0.04	**0.01**	**0.19**
	Body size	− 0.18	0.21	− 0.59	0.23
	*Family specific parameters*				
	Sigma	0.20	0.01	**0.18**	**0.22**
Latency to jump	*Group-level effects (ID)*				
	sd(intercept)	0.10	0.02	**0.07**	**0.15**
	*Population-level effects*				
	Intercept	− 0.12	0.54	− 1.18	0.95
	High frequency call	− 0.00	0.03	− 0.07	0.07
	Low frequency call	0.08	0.03	**0.01**	**0.15**
	Body size	− 0.12	0.18	− 0.49	0.24
	*Family specific parameters*				
	Sigma	0.18	0.01	**0.16**	**0.20**
Speed	*Group-level effects (ID)*				
	sd(intercept)	0.90	0.14	**0.66**	**1.20**
	*Population-level effects*				
	Intercept	− 0.95	3.94	− 8.79	6.87
	High frequency call	0.30	0.18	− 0.05	0.64
	Low frequency call	− 0.31	0.17	− 0.66	0.02
	Body size	0.77	1.36	− 1.94	3.49
	*Family specific parameters*				
	Sigma	0.94	0.05	**0.85**	**1.05**

Estimates and credible intervals (l-95% and u-95%) are presented. A constant transformation has been applied on the three response variables. Significant credible intervals (i.e., non-overlapping zero) are written in bold.

## Discussion

In this study, we used a free-ranging population of the Neotropical poison frog *Allobates femoralis* to investigate whether personality, particularly the personality trait aggressiveness, is related to the degree of plasticity in male territorial behavior. In *A. femoralis*, male reproductive success is directly linked to territory ownership^28^. Loss of a territory to a rival male can result in the complete loss of reproduction, with little chance to compensate in the following season because only ~ 20% of all adults survive until the next reproductive season^28^. Therefore, all individuals should show a high level of territorial aggression towards any intruder to maintain territory ownership, which is corroborated by an absence of a ‘dear-enemy’ effect in *A. femoralis*^49^. But territorial defense is costly^61–63^, and while highly aggressive individuals might have an advantage in repelling competitors, they likely suffer from generally higher energetic expenditures from more aggressive interactions, and particularly costly escalations against competitors they are unlikely to repel. Highly aggressive individuals might also erroneously attack females and thereby loose mating opportunities (pers. Obs. MR and MP; attacks on non-calling robotic frog models have also been observed by^42^). Contrary, less aggressive individuals limit their energy expenditure, thereby increasing their survival, but might be more likely to lose their territory to competitors, even those they would be able to repel. For these reasons, we expected to find within-individual consistency and between-individual differences in territorial defense.

Our results correspond with this prediction and with previous results in *A. femoralis*^30^. We confirmed the existence of a latent variable explaining the covariance of the behaviors measured during a territorial defense test. The repeatability of aggressive behaviors we found was in the range of what has been previously found in *A. femoralis* showing that aggressive behavior measured in a territorial defense context ranged in repeatability from 0.17 to 0.40^30,31^. While these values are slightly lower than the average repeatability found in animal personality studies (R = 0.37, 95%-CI 0.35, 0.38, cf.^64^), they are consistent with previous findings in amphibians^30,31,65^. In our study, the somewhat lower repeatability seems to be driven by a high within-individual variation in the latency to react to the intruder, rather than to low variation between-individual (Fig. 3). In their review, Bell et al. (2009) suggest that ectotherms have lower repeatability than endotherms because ectotherms are more influenced by their environment.

We also observed that all males, regardless of their general aggressiveness level, consistently reduced their response to large intruders (Fig. 2). Individuals showed differences in defense behavior (i.e., personality) and plasticity, but did not adjust their reaction to differently sized intruders depending on their personality. This means that we did not find a link between personality and plasticity in our study. It is possible that the general response whether to engage a territorial intruder or not shows only little plasticity, whereas then the decision to escalate a conflict with an approached intruder to physical combat is highly plastic. In this case, we would expect highly aggressive individuals to physically attack (e.g., wrestle) any intruder immediately, while less aggressive individuals should only attack individuals which they have assessed to be smaller or of similar size, compared to themselves. However, results from a previous study suggest that not speed to approach an intruder but rather experience (e.g., age) influences the likelihood of an attack^46^.

The adaptive value of a link between personality and plasticity might also be conditional on the costs and benefits within the context in which it is measured. For instance, male song sparrows (*Melospiza melodia*) respond more intensely to strangers than to neighbors when their mate is not fertile, but respond similarly to neighbors and strangers when their mate is fertile^66^. In male *A. femoralis*, territories are strongly defended during the breeding (rainy) season^39^. In species maintaining year-round territories with specific mating seasons, we might expect a correlation between personality and plasticity outside of the reproductive period, when fitness expectations are lower. Less aggressive males should be more flexible (e.g., not responding to their neighbors and only responding lightly to strangers), while more aggressive males should be more rigid and be less efficient in differentiating between neighbors and strangers. Likewise, there could be a link between another personality trait (e.g., boldness, exploration) and the level of behavioral plasticity. In *A. femoralis*, future studies should investigate the interplay of personality traits and plasticity across contexts, for instance in periods with low and high reproductive activity.

In our study we wanted to present focal males with simulated intruders at the extremes of the natural size range to elicit clear yes/no responses towards smaller and bigger intruders. Our finding that the body size of the focal male did not affect their response corroborates this notion and implies that the relative size difference did not affect the aggressive response of the focal males in a gradual way beyond reacting plastically to larger *versus* smaller intruders. However, we did not record and measure the calls of our focal males, and therefore could not estimate the body sizes corresponding to the call frequencies of the stimuli we used in our study. While previous studies have shown a correlation between body size and frequency in *A. femoralis*^32,43^, they did not provide the exact regression function in their paper, which would have enabled us to estimate the size of the mimicked intruder we presented. Therefore, we could not include absolute values of size differences in our analysis. In frogs, acoustic contests are a means to avoid escalated physical fights. Assessing an intruder’s body size through its call could help territory holders to judge the likelihood to win such fights. However, there might be other factors influencing the outcome of aggressive territorial interactions.

Overall, our study did not find the expected link between personality and plasticity. Future studies should aim to investigate this link in a range of species and contexts to improve our understanding when it pays off to be plastic and when it is better to be consistent. In most amphibian species, individuals exploit different habitats during their life in which they face different challenges. Therefore, future studies should aim to investigate whether correlations between personality traits and plasticity exist in different life phases and in a variety of contexts.

## Data Availability

The datasets generated and/or analyzed during the current study are available in the Open Science Framework repository: https://osf.io/ue29c/?view_only=14b4a9c4f3fb4724a191149b7fe2bc52.
